# Visual inputs decrease brain activity in frontal areas during silent lipreading

**DOI:** 10.1371/journal.pone.0223782

**Published:** 2019-10-10

**Authors:** Julio Plata Bello, Carlos García-Peña, Cristián Modroño, Estefanía Hernández-Martín, Yaiza Pérez-Martín, Francisco Marcano, José Luis González–Mora

**Affiliations:** 1 Department of Physiology, Faculty of Medicine, University of La Laguna, S/C de Tenerife, Spain; 2 Hospital Universitario de Canarias (Department of Neurosurgery), S/C de Tenerife, Spain; 3 Hospital Universitario de Canarias (Department of Neurology), S/C de Tenerife, Spain; Federal University of Rio Grande do Norte, BRAZIL

## Abstract

**Aim:**

The aim of the present work is to analyze the modulation of the brain activity within the areas involved in lipreading when an additional visual stimulus is included.

**Methods:**

The experiment consisted of two fMRI runs (lipreading_only and lipreading+picture) where two conditions were considered in each one (oral speech sentences condition [OSS] and oral speech syllables condition [OSSY]).

**Results:**

During lipreading-only, higher activity in the left middle temporal gyrus (MTG) was identified for OSS than OSSY; during lipreading+picture, apart from the left MTG, higher activity was also present in the supplementary motor area (SMA), the left precentral gyrus (PreCG) and the left inferior frontal gyrus (IFG). The comparison between these two runs revealed higher activity for lipreading-only in the SMA and the left IFG.

**Conclusion:**

The presence of a visual reference during a lipreading task leads to a decrease in activity in frontal areas.

## Introduction

Lipreading is the ability to visually perceive the speech of others by viewing the movements of their lips, teeth, tongue and jaw [1]. This ability may be considered a complex brain process because it engages sensory, perceptual, memory, cognitive and linguistic elements. Lipreading improves auditory speech discrimination [2] and it is particularly important for the hearing impaired population [2,3]. There is a larger inter-individual variability in lipreading ability (within and across normal hearing and deaf populations) than in auditory speech perception [4]. In this sense, the more skilled lip-readers are able to perceive all levels of speech patterns (from syllables to connected speech) [5]. Understanding how the lipreading ability can improve is essential given that lipreading can substitute for hearing in the education of deaf children [6].

Many brain regions involved in speech production and comprehension are also involved during lipreading. A robust activation in the superior and middle temporal gyrus (STG and MTG), the inferior frontal gyrus (IFG), the inferior parietal lobule (IPL) and the occipito-temporal junction is normally found [1,5,7,8]. Furthermore, there is usually greater activation in the left than the right hemisphere [1,9]. The implication of parietal and frontal regions during lipreading is also supported by the theoretical framework of the mirror neuron system (MNS) [10] and it is also compatible with the longstanding motor theory of speech perception [11]. On one hand, the MNS is formed by a group of regions located in the IPL and IFG bilaterally and they are activated during the execution and the observation of a motor action [12]. Bearing this in mind, considering the speaking language as a sequence of motor actions performed with the mouth and the tongue, IPL and IFG regions are going to be activated during speaking and the observation of speaking [10]. On the other hand, the motor theory of speech perception considered that phonetic information is perceived by detecting the gestures of the speaker [11]. Therefore, both theories are complementary, with the MNS giving an anatomical and functional support to the theory proposed by Liberman (1985).

Furthermore, the Dual Stream model proposed by Hickock and Poeppel (2007) also supports the presence of a lipreading network. Their model suggests a dorsal processing stream including frontal areas that process articulatory movements for the purpose of speech production and perception (IFG and dorsal premotor cortex), as well as a ventral stream formed by posterior areas which parse auditory speech (STG and MTG) and integrate phonology with articulatory movements (at the IPL) [13]. These regions are also known to be engaged even when syllables are presented only-visually [9] though it seems that modality-specific representations exist only to the level of the whole words [5]. Thus, the ventral stream is involved in processing speech signals for comprehension and the dorsal stream is involved in translating acoustic signals into articulatory representations [14].

The brain network involved in speech comprehension is known to be modulated by visual inputs. The recognition of perfectly audible speech is impaired when it is combined with visible facial movements that are incongruent with the acoustic signal [15]. A widely-known example of this is the McGurk effect, in which the auditory perception is modified by a visual input (e.g. an auditory /ba/ dubbed onto a visual /ga/ is heard as /da/) [16]. Using neuroimaging methods, Kislyuk et al (2008) also reported a modulation of the auditory cortex by visual information [17]. The visual modulation of speech perception may generate comprehension problems, mostly in the deaf population. This is especially important in educational and social contexts, where an inadequate speech perception can lead to significant misunderstandings. Therefore, the visual system modulates speech comprehension and this has a social relevance. However, this effect has not been completely studied. For example, what happens when only lipreading is considered (i.e. without auditory information) has not been investigated. This may be important for understanding the modulation of the visual system of lipreading perception in deaf people. In this regard, the presence of other visual stimuli (different from the mouth and tongue movements, e.g. objects) while lipreading may modulate the activity in brain regions involved in lipreading and, consequently, in speech comprehension.

Bearing all the above in mind, the aim of the present fMRI work is to study the modulation of the brain activity within the areas involved in lipreading when an additional visual stimulus is included. The experiment consisted of two fMRI runs: lipreading_only and lipreading+picture (i.e. the additional visual stimulus) with two studied conditions each: oral speech sentences (OSS) and oral speech syllables (OSSY) (i.e. the control condition).

## Methods

### Subjects

Thirty-six healthy, right handed subjects participated in the study (20 women), with an average age of 26.6 (SD = 6.64). All of participants were university students and they have no history of speech, writing and/or reading disability. Written informed consent was explained and signed. The study was approved by the University of La Laguna Ethics Committee, according to the Declaration of Helsinki.

### Data acquisition and processing

Data for the experiment were collected at the Magnetic Resonance for Biomedical Research Service of the University of La Laguna. Functional images were obtained on a 3T General Electric (Milwaukee, WI, USA) scanner using an echo-planar imaging gradient-echo sequence and an 8 channel head coil (TR = 3000ms, TE = 21ms, flip angle = 90°, matrix size = 64 × 64 pixels, 57 slices/volume, spacing between slices = 1 mm, slice thickness = 3 mm). The slices were aligned to the anterior commissure–posterior commissure line and covered the whole cranium. Functional scanning was preceded by 18 s of dummy scans to ensure tissue steady-state magnetization.

A whole-brain three-dimensional structural image was acquired for anatomical reference. A 3D fast spoiled gradient–recalled pulse sequence was obtained with the following acquisition parameters: TR = 10.4 ms, TE = 4.2 ms, flip angle = 20, matrix size = 512 × 512 pixels, .5 × .5 mm in plane resolution, spacing between slices = 1 mm, slice thickness = 2 mm.

After checking the images for artefacts, data were preprocessed and analyzed using Statistical Parametric Mapping software SPM8 (Wellcome Trust Centre for Neuroimaging; http://www.fil.ion.ucl.ac.uk/spm/) and displayed using xjView 9 (http://www.alivelearn.net/xjview/). The images were spatially realigned, unwarped, and normalized to the Montreal Neurological Institute (MNI) space using standard SPM8 procedures. The normalized images of 2 × 2 × 2 mm were smoothed by a full width at half maximum (FWHM) 8 × 8 × 8 Gaussian kernel.

According to the PLOS Data policy the data used in the present manuscript have uploaded in a public repository (https://figshare.com/articles/Lipreading_fMRI_study/9904661).

### Study design

**Fig 1:**
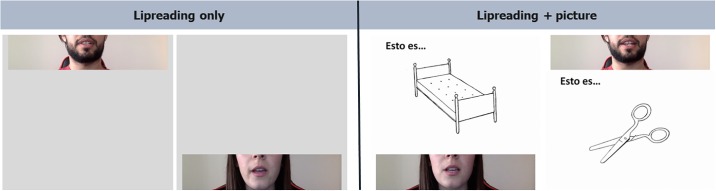
Frames of the video-clips shown in each fMRI run. During the lipreading+picture run, the picture was congruent with the object named during oral speech sentences condition.

Two fMRI runs were performed: lipreading_only and lipreading+picture (Fig 1). The order of the runs was counterbalanced among participants. During each run, participants had to watch a series of four video clips. All video clips showed a male or female speaker whose face was visible only below the nose. It has been shown that facial images showing mouth shape, lips, tongue and teeth position offer potential information about the filter state of the vocal tract [18].

Two conditions were considered in each run: oral speech sentences (OSS) and oral speech syllables (OSSY). The OSS clips consisted of facial movements saying ten short sentences (five sentences in each clip, each sentence lasted 3 seconds) where an object was denominated in Spanish. For example: “This is a helicopter”. The OSSY clips were composed of facial movements repetitively saying ten nonsense syllables during 3 seconds (/la/, /le/, /lo/, /lu/, /pa/, /pe/, /pi/, /ta/, /te/, /to/) (five syllables in each clip).

The videos were projected 12 times for 15 seconds each. OSS and OSSY conditions were presented in a randomized order and there was a 15 second cross fixation (a break with participants watching a black screen with a white cross in the centre) between each condition. In addition, the fMRI run lipreading+picture showed a picture of an object accompanying the speaker video. These pictures were selected from the Boston naming test [19] and they changed every 3 seconds, thus 5 pictures were shown in each video-clip. In the OSS condition, the picture shown coincided with the object named in the video (i.e. when the speaker was saying “this is a helicopter”, a picture of a helicopter was shown).

### Simple T contrasts

A block design in the context of a general linear model was used, for individual subject analyses (first level), to look for differences in brain activity between OSS and OSSY within and between lipreading_only and lipreading+picture runs. The considered contrasts in the analysis were as follows: OSS > OSSY and OSS < OSSY in each run. The first-level contrast images were then used in a random-effects group analysis (second level). Group analysis was performed using the random effect approach, using an ANOVA design and including the age, gender and Edinburgh Handedness Inventory score [20] as covariates. The statistical significance was stablished using the Family Wise Error rate (FWE) = 0.05, with a minimum cluster size of ten voxels.

### Region of interest (ROI) analysis

In addition to the voxel-wise analysis, we also conducted a region of interest (ROI) analysis in regions that have been implicated in lipreading[5]. ROIs of the IFG (Brodman areas [BA] 44 and 45); STG and MTG (BA 21, 22, 41 and 42); and IPL (BA 39 and 40) were created using the WFU Pickatlas software[21]. These images were used for a ROI analysis using MarsBaR 0.44 toolbox (http://marsbar.sourceforge.net/) in the contrasts previously described. The significance within this analysis was a corrected P-value = 0.05.

## Results

### Oral speech sentences (OSS) vs. oral speech syllables (OSSY)

**Fig 2:**
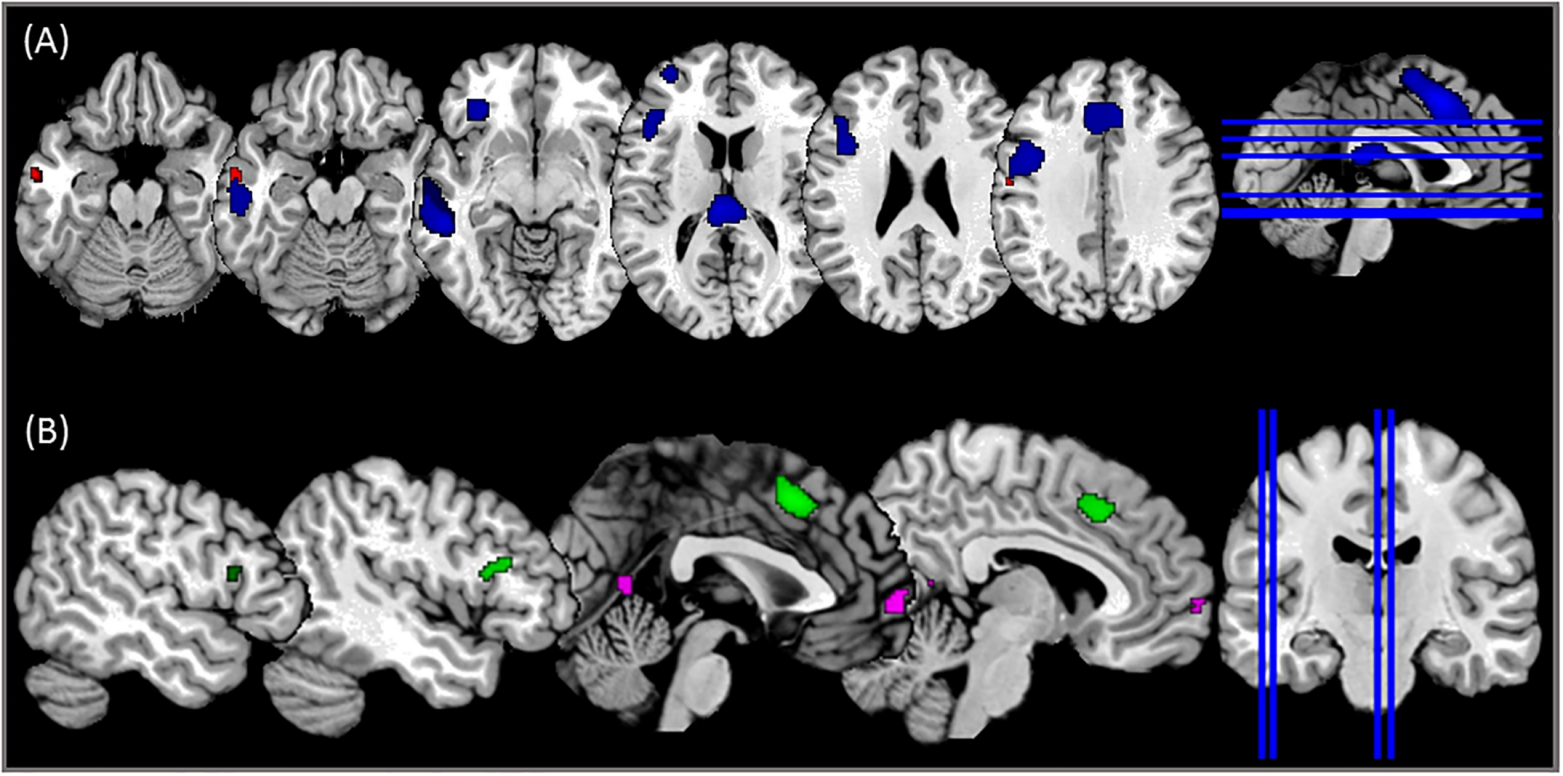
(A) Brain activity during lipreading+picture (OSS>OSSY) (red) and lipreading_only (OSS>OSSY) (blue); (B) Differences in brain activity between lipreading+picture and lipreading only. Clusters in green represent the presence of higher activation for lipreading_only than lipreading+picture; clusters in pink represent the opposite contrast.

During the lipreading+picture run, higher activity in the left MTG (Brodman areas [BA] 21) was identified for OSS than OSSY (Table 1, Fig 2A). The lipreading_only run also showed higher activity in the left MTG in the contrast OSS > OSSY but, additionally, this contrast showed higher activity in the supplementary motor area (SMA), the left precentral gyrus (PreCG), left superior frontal gyrus (SFG) and left IFG (Table 1, Fig 2A). The opposite contrast (OSSY > OSS) did not lead to significant activations in any of the runs with the selected level of significance (FWE = 0.05).

**Table 1:** Activation peaks with their locations for simple T contrasts in each group of patients (FWE = 0.05 at peak level; k = 10 voxels).

Anatomical region	BA	Peak MNI coordinates			t -value	z—value	Num. Voxels
		X	Y	Z			
**Lipreading + picture**: **OSS > OSSY**							
**Left MTG**	21	-60	-18	-10	5.38	5.11	**110**
		-54	-14	44	4.99	4.77	**23**
**Lipreading only**: **OSS > OSSY**							
**SMA**		-2	12	50	7.93	7.18	**1510**
		-6	0	60	6.52	6.08	
**Left MTG**	21	-54	-36	-8	7.40	6.78	**643**
		-60	-20	-10	6.49	6.05	
**Corpus callosum**		-4	-32	18	6.40	5.98	**341**
**Left PrCG**	4	-42	-8	54	6.14	5.76	**954**
		-44	20	22	5.99	5.64	
		-48	-12	50	5.69	5.64	
**Left IFG**	44, 45	-32	26	-8	5.87	5.53	**192**
**Left SFG**		-34	50	20	5.40	5.13	**114**
**Lipreading + picture > Lipreading only** **(OSS > OSSY)**							
**Left mFG**	10	-4	60	6	5.32	5.06	**120**
**Left PosCG**		-2	-62	12	4.80	4.60	**63**
**Lipreading only > Lipreading + picture** **(OSS > OSSY)**							
**SMA**		-2	12	50	6.10	5.73	**457**
		8	18	44	5.40	5.13	
		-12	2	54	4.97	4.75	
**Left IFG**	44, 45	-48	12	18	4.91	4.70	**56**
		-46	20	22	4.81	4.62	

### Lipreading+picture vs. lipreading_only

The comparison of the contrast OSS > OSSY between the two different runs revealed the presence of higher activity in the SMA and the left IFG during lipreading_only, whereas higher activity in the left medial frontal gyrus and the left posterior cingulate gyrus was identified for lipreading+picture (Table 1, Fig 2B).

### ROI analysis

A region of interest (ROI) analysis in regions that have been implicated in lipreading was performed[5]. ROIs of the IFG (BA 44 and 45); STG and MTG (BA 21, 22, 41 and 42); and IPL (BA 39 and 40) in the left hemisphere were created. The ROI analysis showed a significant increase of activity in the primary associative auditory cortex (STG and MTG, BA 21) when the contrast OSS > OSSY was considered in both runs. Furthermore, the left IFG (BA 44 and 45, Broca’s area) showed higher activity during OSS than OSSY when there was no picture and when compared with the other fMRI run (S1 Table).

## Discussion

The present work has studied the differences of brain activity in the lipreading network when the lipreading was accompanied with or without a visual stimulus (i.e. a picture of the object that was being named). The presence of an additional visual stimulus during a lipreading task led to a different pattern of brain activity in frontal areas (left IFG and SMA). This finding will be discussed below.

As described above, the activation of language related areas is highly consistent during lipreading tasks, using either words or syllables [9]. In fact, it is widely accepted that syllable-production related movements elicited a similar pattern of brain activity compared to spoken-words production related movements [5,22]. Here, the syllable lip-reading (OSSY condition) has been used as the control condition and it has been compared with a word/sentence lipreading (OSS condition). Thus, the pattern of brain activity that has been obtained should not be considered as the pattern of activity during a lipreading task, but the pattern of a lipreading task with a specific semantic component (i.e. a sentence naming an object). Bearing this in mind, the presence of the object that was named while lipreading the same object modulated the activity of regions that are normally activated during speech production and comprehension. In this sense, when the picture was absent, higher activity in frontal areas (IFG and SMA) was detected than when the picture was present.

Using a similar paradigm to the present work (i.e. a comparison between lexical [bisyllabic high-frequency words] lipreading vs. non lexical [bisyllabic legal nonwords] lipreading), Paulesu et al (2003), in a positron emission tomography (PET) study, described a similar pattern of brain activation as the present work, with higher activation for the lexical lipreading task (bisyllabic high-frequency words) in the L-IFG, L-MFG, L-PreCG and the R-IFG [22]. The higher implication of frontal regions during the OSS condition than OSSY in the lipreading_only task is assumed by the requirement of further activation in language related areas implicated in access to lexical semantic knowledge [23,24]. Nowadays, it is widely accepted that the left IFG (where Broca’s area is located) is involved in motor speech production as well as speech perception. Phonological processing is a necessary means of accessing lexical and semantic information during visual speech [1]. Thus, the left IFG is not only involved in articulatory-based mechanisms of speech perception, but also has a key role in language comprehension, supporting the information of syntactic and semantic structure [25,26].

However, different activity in frontal areas did not appear when the lipreading task is accompanied with a picture (i.e. the lipreading+picture task). In this respect, the presence of a visual stimulus (i.e. a picture) seems to modulate the activity during a lipreading task, leading to a decrease in frontal activity or an increase in frontal activity during the control condition (i.e. OSSY). This finding may be associated with a different use of the dorsal and ventral pathways of the speech processing system between the studied conditions. As stated above, the dual-loop model makes a distinction between a dorsal and a ventral pathway that anatomically consists of white matter tracts running either above or below the Sylvian fissure [27,28]. During lipreading comprehension, both streams are also involved, but there is a dominance of the dorsal stream, activated in this case, not by acoustic signals, but visual ones (i.e. mouth, lips and tongue movements). The ventral stream seems to be less activated during lipreading tasks when there is no semantic content (i.e. OSSY condition). Nevertheless, when an additional visual stimulus is incorporated (i.e. a picture of an object), the ventral stream is activated by the meaning of this picture and it is strong enough to eliminate the differences that exist between OSS and OSSY conditions when there are no additional visual stimuli. Changing the visual stimuli used here (i.e. a picture) for another visual cue (e.g. picture with parts of the object, a sentence, a word or even letters) would also lead to an activation of the ventral stream, although this activation would be variable according to the cue type. Furthermore, bearing in mind that the pictures that appear during lipreading+picture task were the same either during OSS or OSSY, the information that reached frontal areas via the ventral stream was the same in both conditions. This information activated per se frontal speech areas that are closely tied to the motor plan that has to be executed to produce the same speech movement [29]. The presentation of an incongruent visual stimuli would lead to different results [30].

Although the dual stream model may explain the existence of similar IFG activity in both conditions during lipreading+picture run, it would not explain the absence of SMA differences, which seem to be more associated with the information that flows through the dorsal stream. In any case, connectivity between IFG and SMA has already been described via the corticocortical frontal aslant tract, which allows language production, but not only its elaboration but also its monitoring [31]. Thus, the absence of differences in the activation of the IFG between OSS and OSSY conditions may be related to an activation of the ventral stream that led to the same activation because the stimulus was the same (i.e. object picture). Connections between IFG and SMA may also explain the absence of differences at this level.

Finally, higher activation in the left temporal lobe was identified in the OSS than in the OSSY on both fMRI runs. More specifically the left MTG, consistent with the primary associative auditory region (BA 21), showed this higher activity (Fig 2A). The presence of high level semantic elements during OSS could be the explanation for this higher activity in the left MTG because this area is more strongly recruited when the speech has more linguistic complexity [1]. Therefore, lipreading tasks recruit temporal areas involved in auditory processing (through thalamic connections or direct inputs from the visual cortex [32]) and they can be modulated by the semantic component of the task.

Bearing in mind the findings of the present work, one can make a parallelism between the modulation of lipreading processing by the presence of an additional visual stimulus reported here and the McGurk effect, which consists of a modification of the auditory perception by a visual input [16]. In any case, the identification of a modulatory effect of language perception (in any of their forms) is of great interest, because it allows a better understanding of language processing and, moreover, controlling those factors, one can improve communication. This may be particularly important in patients with difficulties in language processing as well as patients with any auditory impairment. In any case, more studies (preferably those focusing on patients with awake neurosurgical procedures and direct cortical stimulation procedures) are necessary to replicate the findings of the present study and to improve the knowledge about the factors influencing language processing.

## Conclusion

Lipreading perception can be modulated by the presence of a different visual stimulus. During a lipreading task without an additional visual stimulus, higher activation of frontal areas (including IFG and SMA) was identified when observation of speech sentences is compared with observation of speech syllables. However, when an additional stimulus was added, that difference disappeared, and a similar pattern of activity between both conditions was identified.

## Supporting information

S1 Table ROI analysis (statistical significance was considered when corrected p-value was below 0.05). (DOC)
