# Supplementary material for: Visual inputs decrease brain activity in frontal areas during silent lipreading
Source: PLoS One. 2019 Oct 10;14(10):e0223782. doi: 10.1371/journal.pone.0223782 (PMC6786756; doi:10.1371/journal.pone.0223782)
Supplement: S1 Table — (DOC) [file pone.0223782.s001.doc]

**Supplementary table.** **ROI analysis (statistical significance was considered when corrected p-value was below 0.05).**

|  |  | **Lipreading + picture:**  **OSS > OSSY** | **Lipreading + picture:**  **OSSY > OSS** | **Lipreading only:**  **OSS > OSSY** | **Lipreading only:**  **OSSY > OSS** | **Lipreading + picture > Lipreading only** | **Lipreading only > Lipreading + picture** |
| --- | --- | --- | --- | --- | --- | --- | --- |
| **BA 21L** | t-statistic | 4.20 | 1.17 | 5.02 | 1.97 | 0.75 | 1.12 |
|  | Corrected P | **0.000266*** | 0.762292 | **0.000009*** | 0.247308 | 0.942280 | 0.791395 |
| **BA 22L** | t-statistic | 1.33 | -1.33 | 0.55 | -0.55 | 0.44 | -0.44 |
|  | Corrected P | 0.657191 | 0.999999 | 0.976965 | 0.999999 | 0.987986 | 0.999995 |
| **BA 39L** | t-statistic | 1.25 | -0.50 | 1.84 | 2.16 | 1.97 | 0.60 |
|  | Corrected P | 0.713581 | 0.999998 | 0.315807 | 0.164862 | 0.245902 | 0.971090 |
| **BA 40L** | t-statistic | -0.59 | 0.95 | -1.81 | 1.81 | 0.47 | -0.47 |
|  | Corrected P | 0.999999 | 0.872714 | 0.999999 | 0.334714 | 0.985721 | 0.999996 |
| **BA 41L** | t-statistic | 0.47 | -0.47 | -0.43 | 0.43 | 0.63 | -0.63 |
|  | Corrected P | 0.985610 | 0.999996 | 0.999994 | 0.988297 | 0.965438 | 0.999999 |
| **BA 42L** | t-statistic | 0.61 | -0.61 | -1.16 | 1.16 | 1.28 | -1.28 |
|  | Corrected P | 0.968712 | 0.999999 | 0.999999 | 0.768758 | 0.691712 | 0.999999 |
| **BA 44L** | t-statistic | -2.15 | 2.15 | 2.72 | -2.72 | -3.47 | 3.47 |
|  | Corrected P | 0.999999 | 0.168545 | **0.039816*** | 0.999999 | 0.999999 | **0.003884*** |
| **BA 45L** | t-statistic | -2.00 | 2.00 | 2.90 | -2.90 | -3.51 | 3.51 |
|  | Corrected P | 0.999999 | 0.231996 | **0.023369*** | 0.999999 | 0.999999 | **0.003347*** |
